# Anticipated Attack Slows Responses in a Cued Virtual Attack Emotional Sternberg Task

**DOI:** 10.5964/ejop.1896

**Published:** 2021-02-26

**Authors:** Thomas E. Gladwin, Matthijs Vink

**Affiliations:** a Department of Psychology and Counselling, University of Chichester, Chichester, United Kingdom; b Behavioural Science Institute, Radboud University Nijmegen, Nijmegen, The Netherlands; c Centre for Mental Health, Institute for Lifecourse Development, The University of Greenwich, London, United Kingdom; d UMC Utrecht Brain Center, Utrecht University Medical Center, Utrecht, The Netherlands; e Developmental Psychology and Experimental Psychology, Utrecht University, Utrecht, The Netherlands; University of South Wales, Pontypridd, United Kingdom

**Keywords:** emotional Sternberg, threat, inhibition, freezing, cued, attack, anticipation

## Abstract

Threatening stimuli have varying effects, including reaction time (RT) increase in working memory tasks. This could reflect disruption of working memory or, alternatively, a reversible state of freezing. In the current series of experiments, reversible slowing due to anticipated threat was studied using the cued Virtual Attack Emotional Sternberg Task (cVAEST). In this task visually neutral cues indicate whether a future virtual attack could or could not occur during the maintenance period of a Sternberg task. Three studies (N = 47, 40, and 40, respectively) were performed by healthy adult participants online. The primary hypothesis was that the cVAEST would evoke anticipatory slowing. Further, the studies aimed to explore details of this novel task, in particular the interval between the cue and probe stimuli and the memory set size. In all studies it was found that threat anticipation slowed RTs on the working memory task. Further, Study 1 (memory set size 3) showed a decrease in RT when the attack occurred over all Cue Stimulus Intervals (CSIs). In Study 2 a minimal memory set of one item was used, under which circumstances RTs following attacks were only faster shortly after cue presentation (CSI 200 and 500 ms), when RTs were high for both threat and safe cues. Study 3 replicated results of Study 2 with more fine-grained time intervals. The results confirm that anticipation of attack stimuli can reversibly slow responses on an independent working memory task. The cVAEST may provide a useful method to study such threat-induced response slowing.

Emotional reactions may interfere with reflective cognition that depends on undisrupted underlying working memory processes (Hofmann, Friese, & Strack, 2009). Variants of the emotional Sternberg Task provide an opportunity to study interactions between emotional distractors and working memory (Unsworth & Engle, 2007; Wickens, Hyman, Dellinger, Taylor, & Meador, 1986). Trials in the classic Sternberg task (Sternberg, 1966) consist of an encoding phase, a maintenance phase, and a probe phase, requiring the use of working memory (Baddeley, 1992; Kane & Engle, 2003; Petrides, 1996). In the emotional Sternberg Task, emotional distractors can be presented during the maintenance period, which tends to negatively affect performance (Dolcos & McCarthy, 2006; Oei, Tollenaar, Elzinga, & Spinhoven, 2010; Oei, Tollenaar, Spinhoven, & Elzinga, 2009; Unsworth & Engle, 2007); emotional items can also be included in the memory set (Garrison & Schmeichel, 2018).

Effects of emotional distractors could reflect the disruption of working memory processes, but there is an alternative explanation of effects of emotional distractors on reaction time (RT) in particular that draws on the possible role of freezing. This is an evolutionarily preserved defensive response (Blanchard, Blanchard, & Griebel, 2005; Bracha, 2004; Fanselow, 1986) that consists of the simultaneous suppression of movement and strong response preparation for if a fight or flight response needs to be executed (Gladwin, Hashemi, van Ast, & Roelofs, 2016; Roelofs, 2017; Roseberry & Kreitzer, 2017). If a freeze state is induced by an emotional distractor, this could cause inhibition of movement, and hence response slowing. In that case, the slowing effects of a threatening distractor should be reversed by ending the freeze state by presenting a “virtual attack” simulating a stimulus that would require the transition from freezing to fast, energetic responses allowing effective fight or flight behaviour (Bastos et al., 2016; Gladwin et al., 2016; Hashemi et al., 2019; Mobbs et al., 2007; Montoya, Van Honk, Bos, & Terburg, 2015; Nieuwenhuys, Savelsbergh, & Oudejans, 2012).

This possibility was tested in a previous study (Gladwin & Vink, 2018b) using the Virtual Attack Emotional Sternberg Task (VAEST). On some trials neutral faces were presented during the maintenance period of a Sternberg Task. A virtual attack occurred on half such trials, when the neutral face turned angry and appeared to “jump out” at the participant via an increase in size. The question was whether the attack, as a salient emotional distractor, would disrupt working memory and negatively affect performance or, alternatively, act to end a threat-induced inhibitory state. It was found that RTs were slowed when the neutral face was presented but no attack occurred, and this slowing effect was removed by an actual attack. This supported the freeze-release hypothesis: The additional, salient distractor of the attack did not slow RTs further, but ended the slowed state. This reversibility of the slowing effect was the primary interest of the previous study. However, the ability to cleanly interpret the slowing effect of the neutral face was limited as neutral faces slowed RTs even in the absence of attack expectations. Thus, the slowing could not be explained purely in terms of the probability of an attack occurring.

The primary overall aim of the current series of studies was to test the hypothesis that RT slowing would occur after a cue predicting a possible attack and further that this slowing could be removed by an actual attack occurring. If so, this would support the previous results and thereby point to a potentially important alternative explanation for RT slowing due to emotional distractors. Three studies were performed using cued versions of the VAEST (cVAEST) with visually neutral predictive cues to further explore reversible slowing related to threat anticipation. One of the cues was associated with a chance of an attack occurring via a learning procedure. This avoided the above problem with using neutral faces as cues. However, to our knowledge this is a novel variation of the emotional Sternberg task and first steps must be taken in determining whether the expected effects occur but also under which conditions. Therefore, three studies using variations of the task were performed.

In Study 1, the cVAEST was used to determine whether anticipatory slowing and attack-related “release” would occur with a predictive, visually neutral cue rather than the neutral face. In the previous VAEST study as well as in spatial attentional bias tasks using similar anticipatory cues (Gladwin, Möbius, Mcloughlin, & Tyndall, 2019; Gladwin & Vink, 2018a), the interval between the cue and subsequent probe stimulus, the Cue Stimulus Interval (CSI), has a strong impact on effects. Therefore, a range of CSIs was used; these were the same as in the VAEST study. In Study 2 a memory set of only one item was used, to determine whether effects would be found even with such a minimal working memory load. Further, based on the results of Study 1, the CSI around 600 ms was sampled with higher time resolution. Finally, Study 3 added more time intervals to provide a finer-grained view of temporal dynamics. The results of the variations used in the studies are thus of interest for designing future studies; for revealing the time course of effects of anticipated threat; for adding to the knowledge of attentional biases due to anticipatory processing; and for evaluating whether freeze-release effects are robust and replicable.

## Study 1

In the previous VAEST study, slowing due to the presentation of the neutral face (without an attack) was found at all time points, and attacks brought the RT down to a similar level as “safe” trials when no face was presented. Study 1 aimed to determine whether anticipatory slowing would occur with two visually neutral cues, as opposed to the neutral face versus no distractor. Further, the time course of effects may well be different when using predictive cues: it may take more time for a visually neutral threat cue to be identified and for consequent anticipatory responses to occur.

### Method

#### Participants

Participants were recruited online and received either study credits or a small monetary reward (7 dollars) for completing the study, which was performed fully online. Participants were over 18; there were no further inclusion or exclusion criteria for this convenience sample. Participants gave informed consent and the study was performed in line with local ethical guidelines. The total sample consisted of 55 participants who completed the experiment. Data quality checks were performed as explained below to exclude participants with inadequate performance that suggested they were not engaged with the task. This led to the rejection of eight participants. This left 47 participants for analysis (28 males, 19 females) with a mean age of 41 (*SD* = 11.3).

#### Materials

**Figure 1:**
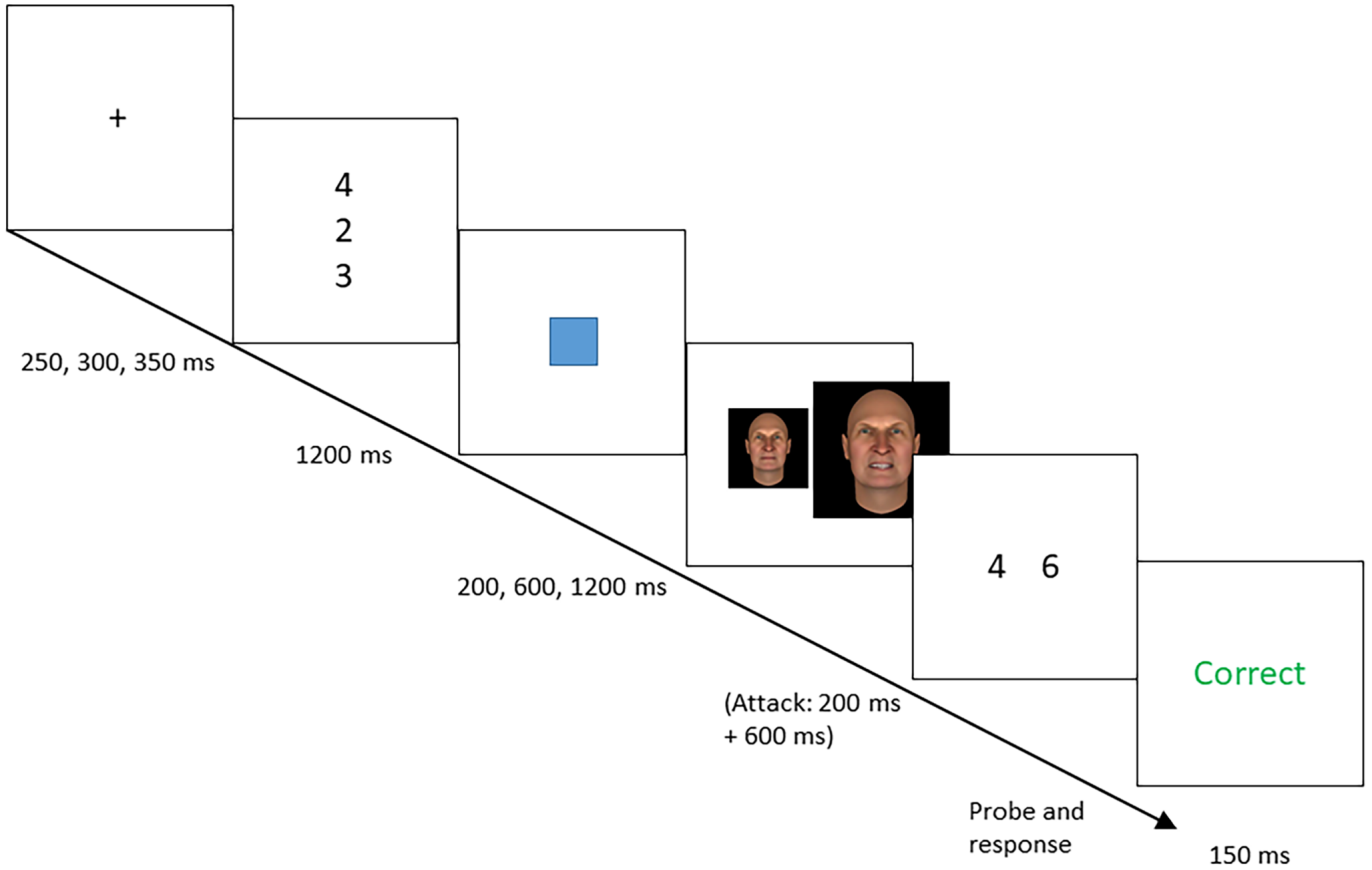
Illustration of the cVAEST *Note*. Trials consisted of encoding, maintenance and probe phases. During the maintenance phase, one of two cues was presented, one of which was never followed by an attack, the other of which was followed by an attack with 50% probability. If an attack occurred, it was inserted between the end of the maintenance phase and the probe. The maintenance phase had a duration (the CSI) of 200, 600 or 1,200 ms before the attack or (on non-attack trials) probe occurred. The attack consisted of an angry face, first presented at a small size for 200 ms, and then at a larger size for 600 ms, creating the effect of a sudden approach. cVAEST = cued Virtual Attack Emotional Sternberg Task; CSI = Cue Stimulus Interval.

The Cued Virtual Attack Emotional Sternberg Task (cVAEST) is illustrated in Figure 1. Trials began with a fixation cross for 250, 300 or 350 ms (all equally likely, as with all further varying duration values). The encoding phase lasted 1,200 ms during which a memory set was presented of three different numbers from 1 to 9, positioned in a vertical column. The maintenance phase had a duration of 200, 600 or 1,200 ms, during which a simple cue was presented in the center of the screen: a blue or yellow square (although this could not be precisely controlled, the square covered around 1 degree visual angle). The attack stimulus never followed one of the cues (the safe cue) and followed the other cue with 50% probability (the threat cue; which color cue was mapped to threat versus safe was randomized per subject). In this task version, an equal number of trials were presented with safe cues, threat cues without an attack, and threat cues with an attack. If a virtual attack occurred, this was added at the end of the maintenance phase. The attack consisted of a 200 ms presentation of a smaller image of an angry face (around 3 degrees visual angle), followed by a 600 ms presentation of a larger image of the face (around 6 degrees visual angle). This created a “jumping-out” effect expected to induce mild threat. Faces were taken from the Bochum Emotional Stimulus Set (BESST; Thoma, Soria Bauser, & Suchan, 2013). Following the maintenance phase or attack, the probe stimulus appeared. This consisted of two different numbers, each from 1 to 9, positioned next to each other. One of the two numbers had been presented in the encoding phase. Participants had to choose which of the numbers that was by press the corresponding left (“F”) or right (“J”) key. The task only continued after a response. The task was programmed in JavaScript, based on the onlineCBM framework (Gladwin, 2017b).

There were three versions or phases of the cVAEST, two of which were used as a learning phase. In all versions, blocks consisted of 32 trials. The first, “100% Attack” version consisted of two blocks. In this version, differently from the other two versions, threat cues were always followed by the attack in order to enhance participants' ability to recognize the cue-threat contingencies. In the second and third version, threat cues were only followed by the attack in 50% of the trials, as described above. The second and third version consisted of two and nine blocks, respectively.

#### Procedure

Participants first performed the 100% Attack task version. They were then asked to specify which of the two cues was never followed by an attack, and which was sometimes followed by an attack. They then performed the second task, followed by the same test on cue-threat contingencies. Finally, they performed the assessment version, followed by the same test. This learning procedure was implemented to increase the number of participants being aware of the cue contingencies, which was used as an inclusion criterion leading to a more consistent sample for analysis (although a proportion of participants are likely to have guessed correctly).

#### Data Analysis

Only the assessment task was analyzed. In preprocessing, the first four trials of the task, the first trial per block, and trials with RTs below 100 ms or above 2,500 ms were removed. Further, for calculation of the RT per condition, trials with RT values that were outliers over the trials within the same condition (absolute *z*-score > 3) were removed. These steps were used to attenuate concerns with noisy data due to online performance, although this does not appear to be consistently worse than in the laboratory (Chetverikov & Upravitelev, 2016).

Within-subject Repeated Measures Analyses of Variance (ANOVAs) with Greenhouse-Geisser correction were used to analyze effects of CSI (200, 600 or 1,200 ms) and Distractor Type (Safe, Threat, Attack). Effects were tested on median RT over accurate trials only, and for mean accuracy over all trials per condition. Median RTs were used to reduce any remaining influence of outliers. Significant effects and interactions were explored using tests performed per level of one of the involved factors and pairwise *t*-tests between levels. Individuals were excluded from analysis who had an RT that was an outlier over participants (absolute *z*-score > 3), an overall accuracy below .9, or an incorrect answer to which cue was associated with threat.

The raw data and analysis scripts are available in Supplementary Materials.

### Results and Discussion

**Figure 2:**
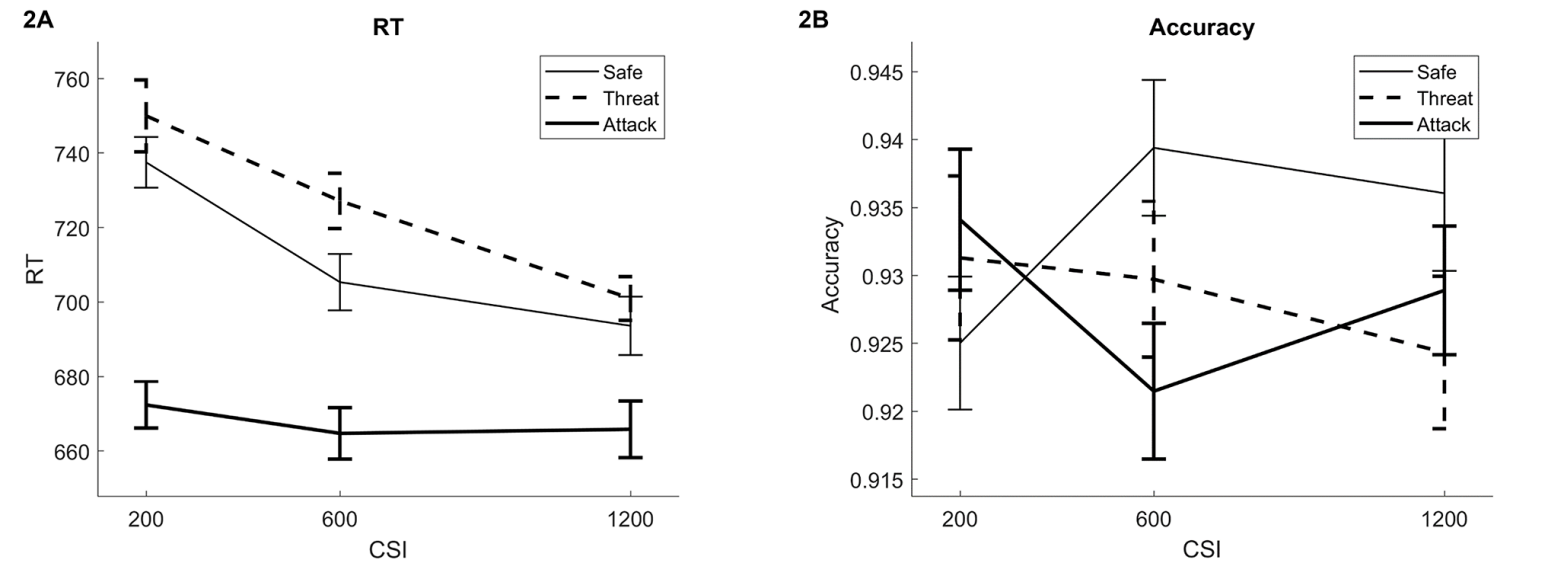
Performance Data on the cVAEST *Note*. Figures 2A and 2B show RT and accuracy data, respectively. The CSIs are plotted on the horizontal axis and the lines show the three trial types: safe cues, threat cues when no attack occurred, and threat cues followed by an actual attack. The figure shows the expected slowing for threat versus safe cues, at the 600 ms CSI especially, and a reduction in RTs when an attack occurs. No significant effects were found for accuracy. cVAEST = cued Virtual Attack Emotional Sternberg Task; CSI = Cue Stimulus Interval; RT = reaction time.

Performance data are shown in Figure 2. There was an effect of Distractor Type, *F*(2, 92) = 45.64, *p* < .001, $\eta_{p}^{2}$ = .50. Tests between levels of this factor showed, first, the expected increase in RTs for Threat versus Safe cues, *t*(46) = 2.96, *p =* .0048, *d* = 0.43 and, second, the expected decrease in RTs for Attacks versus Threat trials, *t*(46) = -8.83, *p* < .001, *d* = -1.28. However, there was also a strong decrease in RTs for Attack versus Safe trials, *t*(46) = -6.029, *p* < .001, *d* = -0.88.

There was also an effect of CSI, *F*(2, 92) = 10.61, *p* < .001, $\eta_{p}^{2}$ = .19, reflecting a decrease in RTs from 200 to 600 ms, *t*(46) = -2.78, *p =* .0079, *d* = -0.40; and a trend for a further decrease from 600 to 1,200 ms, *t*(46) = -1.71, *p =* .095, *d* = -0.25. The interaction between Distractor Type and CSI was not significant, *F*(4, 184) = 2.23, *p =* .082, $\eta_{p}^{2}$ = .046. It was nevertheless further analyzed due to the potential usefulness for further research of information on Distractor Type effects at varying CSIs, and the closeness to significance of the test. The Attack trials had significantly faster RTs than Safe and Threat trials at all CSIs (all *p*s < .033). The slowing effect of Threat cues was only significant at the 600 ms CSI, *t*(46) = 2.22, *p =* .031, *d* = 0.32.

Overall accuracy was .96. There were no significant effects on accuracy.

Thus, the main hypothesis was confirmed: A Threat cue predicting a possible attack slowed responses. Further, this slowing was lost if an attack actually occurred. Unlike the previous VAEST study, however, the RTs following an attack were faster than both Threat and Safe cues, rather than RTs on Attack trials becoming similar to RTs found when there was no threat of attack. It may be that even the Safe cue evoked some anticipatory slowing, although less so than the Threat cue. The slowing effect was strong as a main effect over all time points, but when analyzing the effect per CSI it was only significant at 600 ms; although it should be noted that analyses that are split per CSI involve fewer trials per participant and are therefore expected to be noisier. Nevertheless, the results were taken to suggest focusing on the interval around 600 ms post-cue.

## Study 2

In Study 2, a variation of the task was used to further explore threat-induced slowing. First, the working memory task was simplified: the memory set consisted of only a single element, rather than three. This was expected to reduce the variation in RTs when evaluating the probe stimuli. A further advantage was that less time was needed to present this simplified encoding phase, leading to shorter overall experiment duration. Finally, results of this task design would seem to be of interest theoretically. If clear effects are found even with such a minimal working memory load, this would appear to further support the interpretation of effects in terms of reversible response slowing rather than emotional disruption of working memory processes.

Second, a wider range of attack stimuli was used. Individuals could well differ in what kind of stimuli evoke threat-related processes (Elgersma et al., 2018; Goldin, Manber, Hakimi, Canli, & Gross, 2009; Schulz, Mothes-Lasch, & Straube, 2013). It may therefore be useful to know whether a more varied set of different types of stimuli, versus only variations of faces, can be used as the predicted category. A broader range of stimuli could also decrease habituation, relative to experiencing only variations of the angry faces. Finally, the time period around 600 ms was sampled in more detail by using additional CSIs of 500 and 700 ms. Due to the results of analyses of effects per CSI in Study 1 (only finding a significant slowing at 600 ms), it was predicted that the threat-induced slowing effect would be replicated in the 500, 600, and 700 ms CSI range.

### Method

#### Participants

As in Study 1, a convenience sample of participants was used. Adult participants were recruited online and received either study credits or a small monetary reward for completing the study, which was performed fully online. Participants were over 18; there were no further inclusion or exclusion criteria. Participants gave informed consent and the study was performed in line with local ethical guidelines. Fifty-five participants completed the experiment, of which 15 were rejected in quality checks. This left 40 participants for analysis (28 males, 12 females) with a mean age of 38 (*SD* = 12).

#### Materials

A lower-load version of the cVAEST was used. This was the same as the task in Study 1, with the following changes. There were 25 trials per block in all tasks, and 12 blocks in the assessment task. The memory set consisted of a single number. Safe and Threat cues were increased in size to around 3 degrees visual angle. The Cue-Stimulus Intervals were 200, 500, 600, 700 and 1,200 ms. Attack stimuli could now involve not only angry faces, but also barking dogs, snakes poised to strike, spiders, and gun- and knife-wielding men. An 800 ms response window was included. Finally, Threat and Safe cues were now equally likely, with one-third of Threat cues being followed by an actual attack.

#### Procedure

The same procedure was used as in Study 1, with two learning phases and awareness checks prior to the assessment task.

#### Data Analyses

The same preprocessing steps, quality checks, and statistical analyses were performed as in Study 1. The levels of the CSI factor were now 200, 500, 600, 700 and 1,200 ms. The same three Distractor Type (Safe, Threat, Attack) were used. Further, a paired *t*-test was performed comparing RTs on Threat versus Safe cues averaged over the 500, 600 and 700 ms CSIs.

### Results and Discussion

**Figure 3:**
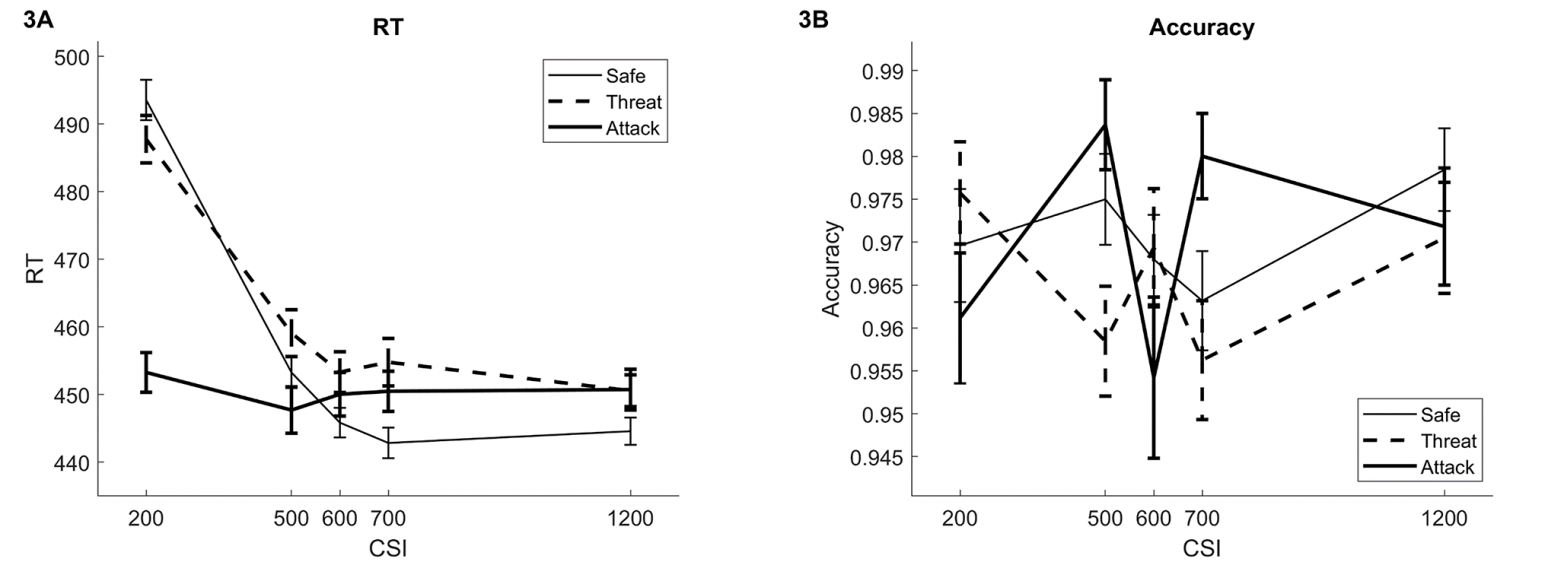
Performance Data on the Low-Load cVAEST (Study 2) *Note*. Figures 3A and 3B show RT and accuracy data, respectively. The CSIs are plotted on the horizontal axis and the lines show the three trial types: safe cues, threat cues when no attack occurred, and threat cues followed by an actual attack. The figure shows the expected slowing for threat versus safe cues and a reduction in RT when an attack occurs following a threat cue. No significant effects were found for accuracy. cVAEST = cued Virtual Attack Emotional Sternberg Task; CSI = Cue Stimulus Interval; RT = reaction time.

Performance data are shown in Figure 3. The main result was that the expected slowing following Threat versus Safe cues over the 500, 600 and 700 ms CSIs was found, *t*(40) = 8.97, *p* < .001, *d* = 1.42.

Further, for RTs, there was an effect of Distractor Type, *F*(2, 78) = 9.5, *p* = .00078, $\eta_{p}^{2}$ = .20, due to the overall Threat versus Safe slowing, *t*(39) = 3.10, *p* = .0035, *d* = 0.49, and faster responses following an Attack versus Threat, *t*(39) = -3.86, *p* = .00042, *d* = -0.61. A trend for faster responses following an Attack versus Safe was found, *t*(39) = -2.02, *p* = .050, *d* = -0.32. Distractor Type and CSI showed an interaction, *F*(8, 312) = 15, *p* < .001, $\eta_{p}^{2}$ = .28, in line with visual inspection of the time courses of RT. The effects of Attack versus Threat and Attack versus Safe were significant at CSI 200 ms (*p* < .001) only. The Threat versus Safe slowing effect was only significant at CSI 700 ms (*p* = .0070) but was near significance at all CSIs above 200 ms (*p*s < .063). There was a main effect of CSI, *F*(4, 156) = 46.19, *p* < .001, $\eta_{p}^{2}$ = .54. This was due to the significant decreases in RT from 200 to 500 ms (*p* < .001).

Overall accuracy was .97. There were an interaction between Distractor Type and CSI, *F*(8, 312) = 2.6, *p* = .012, $\eta_{p}^{2}$ = .063. This was due to a decrease in accuracy for Attack versus Threat trials at CSI 500 and 700 ms (*p*s < .003).

Thus, as expected, the threat-induced slowing found in Study 1 was replicated in the 500 – 700 ms CSI range of interest. The occurrence of an attack decreased RTs but only early in the CSI; RTs following both cue types subsequently decayed over time even without an attack, while RTs on attack trials remained around the same level. The time course of RTs suggested that overall cue-related slowing decreased to a baseline level, reached around 600 ms.

## Study 3

In Study 3, the same task as in Study 2 was used with additional CSIs to better observe the RT time course. The CSI range of 500 – 700 ms appeared to be of particular interest, but the edges of this period were not sampled in Study 2. This CSI range sampled with a 100 ms time steps was therefore extended from 400 to 800 ms. Knowledge of the time course is of methodological importance for future studies aiming to target the most relevant CSIs. More detailed information on the time course of effects, focusing on relevant time ranges, could also be of interest to models of cognitive and emotional processes focusing on temporal dynamics, such as the iterative reprocessing model (Cunningham, Zelazo, Packer, & Van Bavel, 2007) and the R3-reflectivity model (Gladwin & Figner, 2014; Gladwin, Figner, Crone, & Wiers, 2011). From the perspective of such models, it is essential to build up knowledge of how different cognitive processes or representations are more strongly activated at different points in time. The current more detailed exploration of the time course of response slowing provides a foundation for further work in, for example, clinical populations with possibly abnormal temporal dynamics.

### Method

#### Participants

Participants were recruited online and received either study credits or a small monetary reward for completing the study, which was performed fully online. Participants were over 18; there were no further inclusion or exclusion criteria. Participants gave informed consent and the study was performed in line with local ethical guidelines. Fifty-four participants completed the experiment, of which 14 were rejected in quality checks. This left 40 participants for analysis (24 males, 16 females) with a mean age of 40 (*SD* = 10.0).

#### Materials

The cVAEST variant was the same as the task in Study 2, with Cue-Stimulus Intervals of 200, 400, 500, 600, 700, 800 and 1,200 ms.

#### Procedure

The same procedure was used as in Study 1 and 2, with two learning phases and awareness checks prior to the assessment task.

#### Data Analyses

The same preprocessing steps, quality checks, and statistical analyses were performed as in Study 2. The levels of the CSI factor were now 200, 400, 500, 600, 700, 800 and 1,200 ms. The same three Distractor Type (Safe, Threat, Attack) were used. Further, a paired *t*-test was performed comparing RTs on Threat versus Safe cues averaged over the 600, 700, 800 and 1,200 ms CSIs, to represent the time points at which threat-induced slowing was expected based on Study 2.

### Results and Discussion

**Figure 4:**
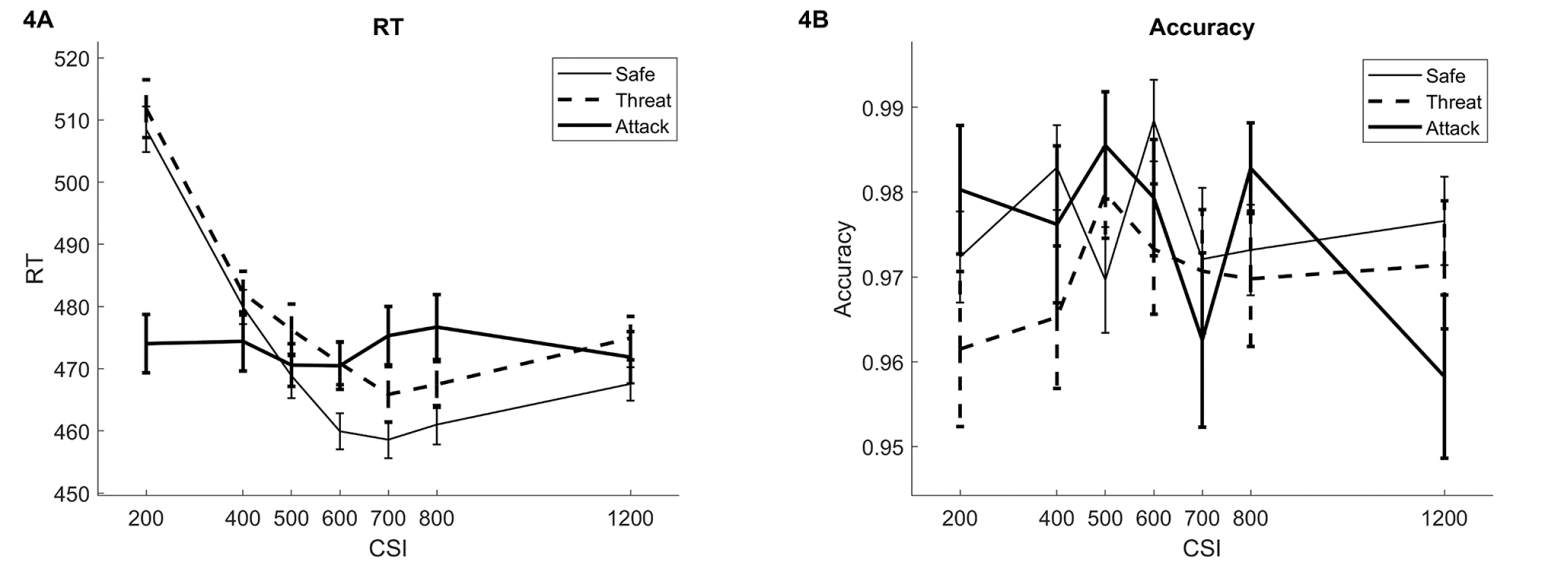
Performance Data on the Low-Load cVAEST (Study 3) *Note*. Figures 4A and 4B show RT and accuracy data, respectively. The CSIs are plotted on the horizontal axis and the lines show the three trial types: safe cues, threat cues when no attack occurred, and threat cues followed by an actual attack. The figure shows the expected slowing for threat versus safe cues and a reduction in RT when an attack occurs following a threat cue. No significant effects were found for accuracy. cVAEST = cued Virtual Attack Emotional Sternberg Task; CSI = Cue Stimulus Interval; RT = reaction time.

Performance data are shown in Figure 4. The expected slowing following Threat versus Safe cues was found, *t*(39) = 2.45, *p* = .019, *d* = 0.39.

For RTs, there was a significant interaction between Distractor Type and CSI, *F*(12, 468) = 7.9, *p* < .001, $\eta_{p}^{2}$ = .17. Attack trials were faster than Threat and Safe trials at CSI 200 ms only (*p* < .001), but were slower that Safe trials at CSI 600, 700 and 800 ms (*p*s < .040). The Threat versus Safe slowing effect was only significant at CSI 600 ms (*p* = .0311). There was a main effect of CSI, *F*(6, 234) = 29.83, *p* < .001, $\eta_{p}^{2}$ = .43. This was due to the significant decreases in RT from 200 to 600 ms (*p* < .039).

Overall accuracy was .97. There were no significant effects on accuracy.

Thus, as expected, the threat-induced slowing found in Study 1 and, more closely, Study 2 was replicated. The occurrence of an attack again decreased RTs only early in the CSI. Over later CSIs, Attack trials were slower than Safe trials.

## General Discussion

The current studies aimed to determine whether cued anticipation of a virtual attack would slow responses on a working memory task. This was confirmed, the data furthermore indicating that this threat-related slowing effect requires some time to develop. Differences between Threat and Safe cues appeared from around 600 CSI, when RTs decayed to lower levels following Safe than Threat cues. While the task was optimized to compare Threat and Safe cues, actual attacks were found to have varying effects on RT, appearing to result in a relatively stable level regardless of CSI; importantly, attacks did systematically reduce RTs shortly after cue presentation, when RTs on both types of non-attack trials were high.

The current results demonstrate, for the first time, anticipatory slowing on a working memory task caused by visually neutral cues predicting an attack rather than actual presentation of threatening stimuli. This was predicted based on the broad literature on freezing and on the previous VAEST study. Of both theoretical and methodological importance, the slowing results contrast with effects of threatening cues in various other tasks, in which responses tend to become faster and more impulsive when threatening cues are presented (De Houwer & Tibboel, 2010; Gladwin, 2017a; Hartikainen, Siiskonen, & Ogawa, 2012; Hashemi et al., 2019; Nieuwenhuys et al., 2012; van Peer, Gladwin, & Nieuwenhuys, 2019; Verbruggen & De Houwer, 2007). This apparent contradiction can be resolved by the duality of the freezing response, which involves both strong response preparation as well as inhibition of movement (Roelofs, 2017; Roseberry & Kreitzer, 2017). One feature of the cVAEST that may be essential in inducing inhibition rather than impulsivity is that participants are performing a threat-irrelevant working memory task, unlike tasks in which responding is based on a simple stimulus-response mapping. Further, there was no performance-contingent aspect to the threat, as is the case in tasks in which an aversive stimulus occurs when performance is inadequate. That is, the attack could not be avoided, possibly leading to a non-preparatory, passive form of freezing, as opposed to active response preparation under simultaneous inhibitory control (Gladwin et al., 2016). This distinction between active versus passive forms of freezing appears to be an important consideration for future research on effects of threat (Bracha, 2004; Roelofs, 2017).

The results partially confirmed the expected “release” effect of the actual occurrence of an attack. At longer CSIs and with a simple task, RTs on non-attack trials became faster than on attack trials, suggesting a disruptive effect of the attack stimulus. However, there was a strong reduction in RT following attacks at a short CSI when responses were relatively slow following either cue, possibly reflecting an orienting component of freezing (Campbell, Wood, & McBride, 1997). The current results thus confirm that response slowing in the emotional Sternberg task is reversible under some conditions. This suggests a possibly important re-interpretation of response slowing by emotional distractors. If it were the case that slowing reflected disruption of working memory by the cues, then this would not be expected to be reversed by an attack. In contrast, the data at short CSIs fit the freeze-release pattern, in which slowing does not reflect working memory disruption but a transient, possibly inhibitory state affecting response execution.

The current study had a number of limitations. First, the tasks involved, by design in Study 2 and 3, low or very low working memory load. Higher working memory loads may be interesting to explore, although the use of low loads does not appear to affect the current conclusions and it should be noted that higher loads could increase noise and that increasing working memory load could suppress emotional effects (Van Dillen, Heslenfeld, & Koole, 2009). Second, while more fine-grained exploration of the range of CSIs allows study of the more precise time course of differences between safe versus threat cues, this reduces the number of trials per condition per participant. Future studies could consider using multiple sessions to acquire more trials without making the task duration longer and hence more fatiguing. Third, it would be interesting to study associations between threat-induced slowing and psychiatric symptoms in larger and/or clinical samples. One interpretation of the results is that they reflect elements of the freezing response, which is involved in disorders (e.g., Hagenaars, Stins, & Roelofs, 2012). Fourth, there are many variations of the task that could be studied in future research, beyond the scope of these first studies. For instance, we make no claim that the event terminating the response inhibition must necessarily involve a threatening attack of the type used in the current studies; perhaps positively valenced events could have similar releasing effects. Note that while freezing is evolutionarily related to threat, it consists of more general underlying processes such as response preparation and inhibition that are not logically exclusive to the context of threat. Other task variants may be more suited to studying the “release” effect of attacks, in particular those in which non-visual attacks are used such as electric shock or loud noise. Such stimuli could also have far shorter time durations than the attack stimulus and allow closer comparability of attack versus non-attack cue types in terms of the timing of the probe stimuli. Tasks focusing on attacks could also study the Attack-Probe Interval in a similar manner as the CSI in the current tasks: It may be the case that effects of attacks show a similar decay following an initial RT increase. Finally, we acknowledge that the interpretation of effects in terms of freezing must be tentative, being based only on behavioural measures. Psychophysiological or neuroimaging measures could provide additional evidence to test this interpretation by considering, for example, bradycardia, body sway and activation of freeze-related brain regions (Hashemi et al., 2019; Hermans, Henckens, Roelofs, & Fernández, 2012; Roelofs, Hagenaars, & Stins, 2010).

In conclusion, visually neutral cues signaling the possibility of an attack were found to slow responses on a concurrent working memory task. The current study thus expands and supports prior results on the freeze-release pattern of effects of threat-related distractors on RT in such tasks and complements the literature on different effects of threatening stimuli and predictive cues. The cVAEST may be an interesting method to study threat-induced response inhibition.

## Data Availability

Data for this article is freely available (see Gladwin & Vink, 2021).

## Notes

The following Supplementary Materials for Study 1, Study 2, and Study 3 are available via the PsychArchives repository (for access see Index of Supplementary Materials below):

- Raw data.
- Analysis scripts.
